# The Effect of On-Pump and Off-Pump Bypass Operations on Oxidative Damage and Antioxidant Parameters

**DOI:** 10.1155/2017/8271376

**Published:** 2017-12-17

**Authors:** Ayse Dogan, Fevzi Sarper Turker

**Affiliations:** ^1^Department of Physiotherapy and Rehabilitation, Bitlis Eren University Health High School, 13 000 Bitlis, Turkey; ^2^University of Health Sciences Elazığ Training and Research Hospital Cardiovascular Surgery, 23 000 Elazığ, Turkey

## Abstract

**Objective:**

The aim of the study is to determine the oxidative status in on-pump and off-pump coronary artery surgery and contribute to possible surgical choices in clinical practices in accordance with the information obtained as a result of this study.

**Methods:**

52 patients undergoing open heart surgery (26 patients in on-pump group and 26 patients in off-pump group) were included in the study. MDA, GPx, GSH, CAT, and SOD were investigated in blood samples.

**Results:**

In the on-pump group, it was determined that there were a significant increase in MDA level in the peroperative period compared to the preoperative and postoperative periods and a significant increase in GSH level in the postoperative period than in the preoperative period. Additionally, while there was a significant decrease in CAT activity in the postoperative period than in the peroperative period, there was a statistically significant increase in SOD enzyme activity in the postoperative period compared to the preoperative and peroperative periods. A statistically significant increase was observed in SOD enzyme activity in the postoperative period in on-pump compared to off-pump group.

**Conclusion:**

It is thought that this oxidative damage can be suppressed by administering a suitable antioxidant supplement in the preoperative and peroperative periods among patients undergoing the on-pump operation.

## 1. Introduction

Free radicals are relatively reactive molecules derived from oxygen, nitrogen, or sulphur molecules. Since these radicals are usually derived from oxygen, these molecules are called as reactive oxygen molecules (reactive oxygen species/ROS) [1, 2]. ROS is the products of normal metabolism such as aerobic metabolism in mitochondria in ATP production. However, being generated under normal conditions, ROS is scavenged by the cells' own defense mechanisms against ROS. Enzymatic and free radical scavenging activities are among these defense mechanisms. In a normal metabolism, the balance between ROS production and the defense mechanisms against ROS prevents the cell injury. However, ROS levels may significantly increase and as a result, the injury may be observed in numerous cellular molecules such as lipids, proteins, and DNA [1, 2]. Superoxide anion (O_2_^−•^), hydrogen peroxide (H_2_O_2_), hydroxyl radical (^•^OH), and peroxynitrite (ONOO^−^) are the essential ROS types leading to oxidative damage in the heart [3]. Open heart surgeries with cardiopulmonary bypass cause oxidative stress. This is closely associated with excess ROS production [4]. Oxidative reaction leads the damaged cell function and may increase complications during or after coronary artery bypass grafting (CABG) surgery [5]. Aortic cross-clamping causes ischemia-reperfusion damage by increasing stress during the operation [6]. Oxidative stress indicators increasing in the postoperative period such as xanthine oxidase, nuclear factor kappa B, and nicotinamide adenine dinucleotide phosphate affect on pathogenesis of postoperative atrial fibrillation (POAF) [7]. Furthermore, the increased production of ROS and superoxide anion is associated with the atrial nicotinamide adenine dinucleotide phosphate oxidase and this highly results in the risk of development of POAF [8].

Atherosclerotic coronary artery diseases are associated with inflammation and oxidative stress before the surgical intervention. Furthermore, cardiac surgical intervention may rise the possibility of diabetes, renal, and lung diseases induced by oxidative stress as well as excessive redox formation in the patients who underwent the surgical intervention [3]. During the surgical intervention, cardiopulmonary bypass (CPB), ischemia, and reperfusion may cause significant myocardial stress. This stress may caused by proinflammatory agents and also the protein, lipids, and DNA damage associated with the formation of ROS [9]. Off-pump coronary artery bypass (OPCABG) technique is an evaded situation due to hemodynamic instability in a beating heart during revascularization. This complication may decrease as a result of development of new stabilization tolls, increased experiences of the surgical team, and a better hemodynamic management during the operation together with the technological development [10, 11]. When OPCABG technique was compared with CABG in some studies, it has been determined that less lipid peroxidation damage occurred [12]. Also in their study, Matata et al. observed that protein carbonylation levels were lower in the patients, to whom OPCABG technique was applied, compared to CABG [13]. Antioxidant molecules may degrade by directly reacting with reactive radicals. Thus, they can be converted into new less active, long-lasting, and less dangerous free radicals. These new free radicals can also be neutralized by other antioxidants or mechanisms [3]. It is a proper strategy to remove the effect of ROS through the combined use of antioxidants such as propofol, L-arginine, and N-acetylcysteine with intravenous infusion or cardioplegia during CPB [3]. Being a closed CPB system associated with decreased surface area, mini-CPB decreases priming volume, elimination of cardiotomy suction, and prevention of air-blood contact [14]. Mini-CPB decreases the nonendothelium surface and the activation of coagulation cascades associated with the red blood cells as well as the fibrinolytic and proinflammatory activities. Also, compared to the patients undergoing operations via traditional methods, serum concentration of MDA tends to decrease through the use of mini-CPB and allantoin/urate ratio that is an oxidative stress marker [15].

Previous studies revealed primarily the excessive ROS production in coronary artery bypass operations [16]. Additionally, when the heart valve patients are compared with coronary patients and surgery patients are compared with the healthy individuals, limited data are obtained about the NO synthesis route and various components of oxidant/antioxidant balance in the circulation [17]. The aim of the present study is to determine the oxidative status in on-pump and off-pump coronary artery surgery and to make contribute to the possible surgical choices in clinical practices in accordance with the information obtained as a result of this study.

## 2. Material and Methods

### 2.1. Population of Study

A total of 52 patients undergoing open heart surgery (26 patients in the on-pump group (5 women and 21 men) and 26 patients in the off-pump group (13 women and 13 men)) were included in the study. All the patients included in this study underwent elective cardiopulmonary bypass. They were informed about the study, and they signed the information consent form stating that they were voluntary to participate in the study. The present study protocol was reviewed and approved by the ethics committee of Firat University (Reg. number 2015015).

In the present study, blood was drawn from each patient 3 times: before the surgical intervention (preoperative period), at the end of the surgical intervention (peroperative period), and approximately 24 hours after the surgical intervention (postoperative period). Malondialdehyde (MDA), an oxidative damage indicator, and the antioxidant indicators which were glutathione peroxidase (GPx), reduced glutathione (GSH), catalase (CAT), and superoxide dismutase (SOD) were examined as the biochemical parameters in these blood samples.

### 2.2. Surgical Technique

All patients were managed by the same surgical and anesthetic team in the same operation room. Six-channel electrocardiogram (ECG) and noninvasive arterial pressure monitorization were applied to the patient taken to the operating table. Before anesthesia induction, radial artery catheter was inserted under local anesthesia and preoperative blood samples were taken together with the baseline blood gas and invasive pressure monitoring was performed. Anesthesia induction was performed by administering 100 mg lidocaine intravenous (i.v.), 300 mg magnesium i.v., 100 *μ*gr fentanyl i.v., 0.60–1.2 mg/kg esmeron i.v., and 2 mg/kg propofol i.v. Central venous cannula and urine catheters were inserted in the postanesthesia period. Maintenance of general anesthesia was performed by adding 20 mg esmeron and 100 *μ*gr fentanyl i.v. in the oxygenator reservoir every 30 minutes.

While propofol infusion (1%) was performed as 20 ml/hour i.v. out of CPB, it was reduced to the infusion dose of 10 ml/hour i.v. during CPB. A median sternotomy was performed to all the patients. The heparin of 350–400 unit/kg was administered to the left internal mammary artery (LIMA) prior to cannulation. This was followed by routine aortic and right atrial cannulation. Membrane oxygenators and moderate systemic hypothermia were used to carry out cardiopulmonary bypass (CPB). Myocardial protection was achieved by using antegrade mild hypothermic blood cardioplegia (32°C) and repeated every 20 minutes. Cold blood cardioplegia was prepared by adding 2 mmol/lt magnesium sulphate, 5 mmol/lt potassium chloride, and 1.6 gr/1000 cc sodium bicarbonate in every 1000 cc blood taken from the reservoir. Activated clotting time was maintained for >400 sec during the procedure. During the procedure, mean blood pressure was kept at 60 mmHg and over. All the proximal anastomoses of saphenous vein were performed via cross-clamp by using a single clamp technique. Air was removed from the proximal anastomoses, and cross-clamp was removed. After sufficient cardiac performance was provided, the pump flow was reduced and CPB was ended. The heparin was neutralized with protamine at the ratio of 1 : 1.3 for 10 minutes after CPB.

In the patients undergoing off-pump operation, the preparation of the patient and anesthesia induction were performed in the same way. After LIMA was harvested and heparin of 80–100 unit/kg was administered following median sternotomy, dose adjustment was set for the active clotting time (ACT) of 250. Primarily, proximal graft anastomoses of saphenous vein were performed by using the side clamp. Then, distal coronary anastomoses were performed by intracoronary shunt and a stabilizer (Estech hercules). First, the left anterior descending (LAD) artery was filled with blood by LIMA or saphenous vein graft. Then, anastomoses of other coronary arteries were performed. Circumflex artery and branches were not made bloody in none of the patients in this study. Esmelol (brevibloc premix 10 mg/250 ml) infusion was started in order to have the heart rate as 70 beats per minute and the blood pressure over 60 mmHg. Heparinized state was reversed by using protamine and closure administered as routine.

After the operation, all on-pump and off-pump patients were followed up in the intensive care unit. Second (peroperative) blood samples were taken from the radial artery catheter together with the blood gas once the patients were taken to the intensive care unit. Third (postoperative) blood samples were taken approximately 24 hours after the end of operation when the intensive care follow-up of the patient continued. All the samples were sent to the laboratory as soon as possible, and they were properly prepared and kept.

### 2.3. Sample Collection

Once blood samples were taken in two heparin-containing experimental tubes by means of the cannula inserted in radial artery, they were taken to the laboratory for invasive blood pressure monitoring. While one of the heparinized bloods was used as the full blood, the other heparinized blood was centrifuged at 3000 rpm for 5 minutes and its plasma was separated and then irrigated three times by using physiological saline solution. Then, it was kept at the deep freezer at −80°C before biochemical analyses.

### 2.4. Biochemical Analyses

#### 2.4.1. Lipid Peroxidation

The MDA assay in plasma was carried out based on the method of Placer et al. [18] with slight modifications. The MDA formed a pink complex with thiobarbituric acid (TBA), and the absorbance read was 532 nm. The plasma MDA content was expressed as nmol/ml.

#### 2.4.2. GSH Level

Being determined in accordance with the method of Chavan et al. [19] the GSH contents were expressed as *μ*mol/g Hb.

#### 2.4.3. CAT Activity

The Sun [20] method was used to measure CAT activity. The degradation rate of H_2_O_2_ by CAT was spectrophotometrically measured by means of H_2_O_2_ absorbing light at 240 nm wavelength. CAT activity was calculated as katal/g Hb.

#### 2.4.4. SOD Activity

The SOD enzyme activity was measured based on degradation of nitroblue tetrazolium (NBT) by the superoxide radical, which was produced by the xanthine-xanthine oxidase system. The blue-colored formosan obtained at the end of the reactions maximally absorbed at 560 nm [21]. The SOD enzyme activity was calculated as U/g Hb.

### 2.5. Statistical Analysis

Statistical analysis was carried out through the SPSS package program (15.0 for Windows). The paired *t*-test was used to assess on-pump data and off-pump data in themselves in terms of oxidative damage and antioxidant parameters. The independent sample *t*-test was used to compare the data of the on-pump and off-pump groups in terms of oxidative damage and antioxidant parameters. Also, the Mann–Whitney *U* test was used to evaluate the routine biochemistry data of the on-pump and off-pump groups in themselves. The value of *p* < 0.05 was accepted as statistically significant. All of the results were shown as mean ± standard error mean (SEM) for parametric tests and median for nonparametric test.

## 3. Results and Discussion

**Figure 1:**
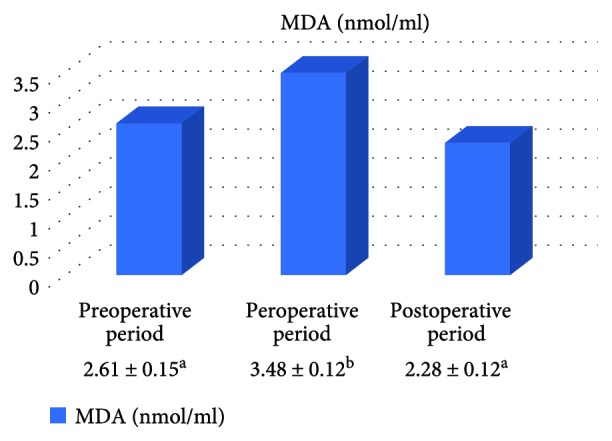
Statistical comparison of oxidative parameter (MDA) in the on-pump group in the preoperative, peroperative, and postoperative periods.

**Figure 2 fig2:**
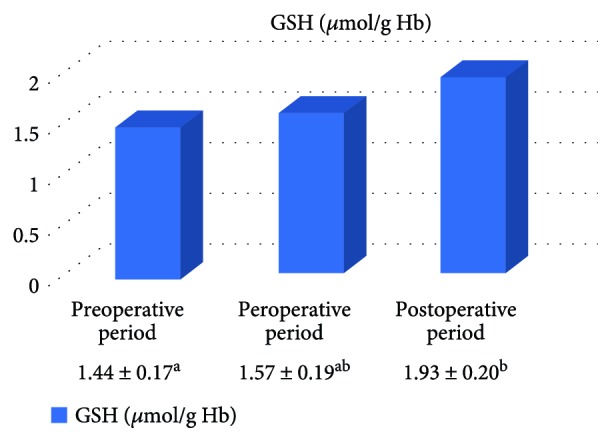
Statistical comparison of antioxidant parameter (GSH) in the on-pump group in the preoperative, peroperative, and postoperative periods.

**Figure 3 fig3:**
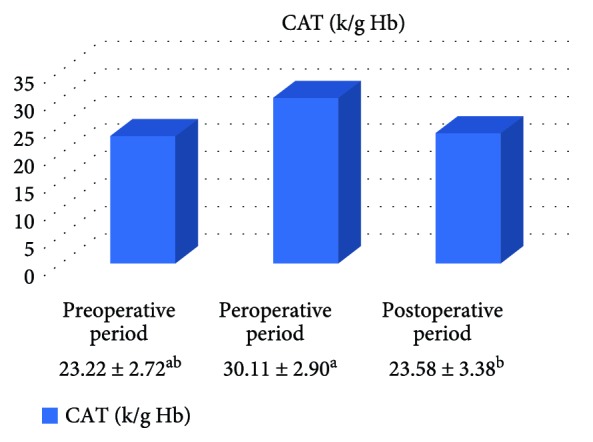
Statistical comparison of antioxidant parameter (CAT) in the on-pump group in the preoperative, peroperative, and postoperative periods.

**Figure 4:**
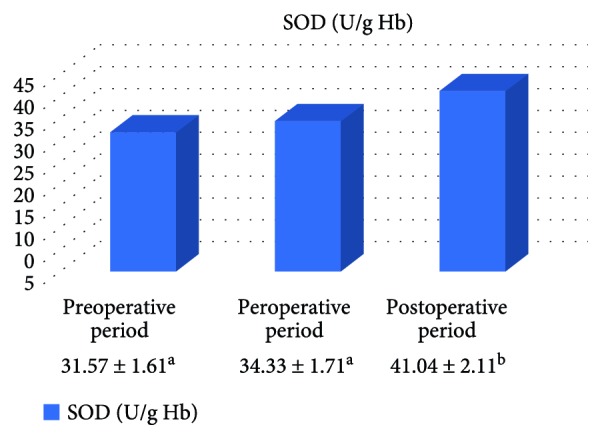
Statistical comparison of antioxidant parameter (SOD) in the on-pump group in the preoperative, peroperative, and postoperative periods.

In the on-pump group, a significant increase was observed in MDA level in the peroperative period compared to the preoperative and postoperative periods and in GSH level in the postoperative period compared to the preoperative period. While there was a significant decrease in CAT activity in the on-pump group in the postoperative period compared to the peroperative period, a statistically significant increase was determined in the SOD enzyme activity in the postoperative period compared to the preoperative and peroperative periods (Table 1, Figures 1 [Fig fig2] [Fig fig3]–4).

**Figure 5:**
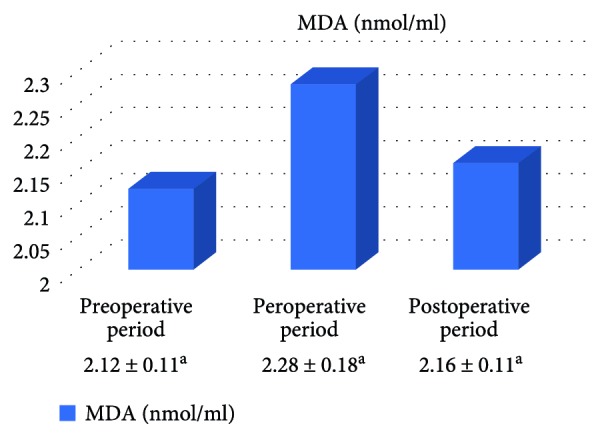
Statistical comparison of oxidative parameter (MDA) in the off-pump group in the preoperative, peroperative, and postoperative periods.

**Figure 6 fig6:**
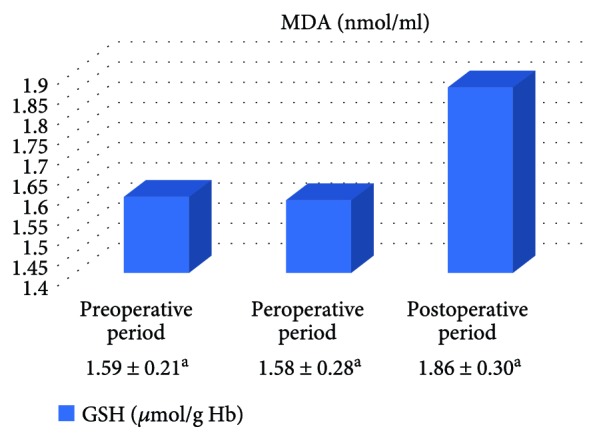
Statistical comparison of antioxidant parameter (GSH) in the off-pump group in the preoperative, peroperative, and postoperative periods.

**Figure 7 fig7:**
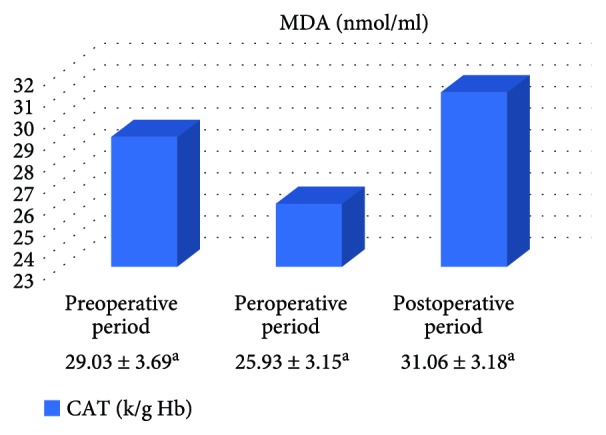
Statistical comparison of antioxidant parameter (CAT) in the off-pump group in the preoperative, peroperative, and postoperative periods.

**Figure 8:**
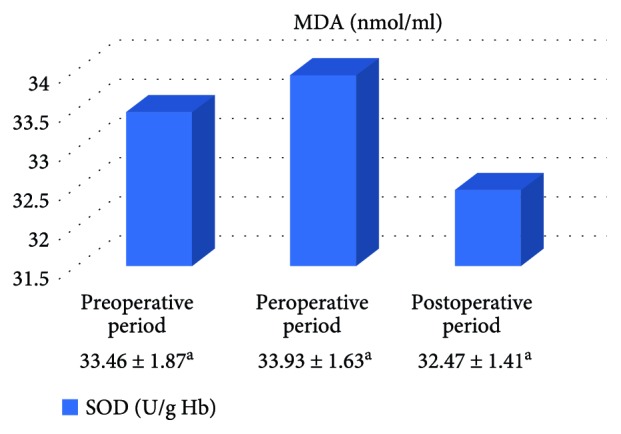
Statistical comparison of antioxidant parameter (SOD) in the off-pump group in the preoperative, peroperative, and postoperative periods.

In the off-pump group, no statistically significant difference was observed between CAT and SOD activities and between MDA and GSH levels (Table 2, Figures 5 [Fig fig6] [Fig fig7]–8).

**Figure 9:**
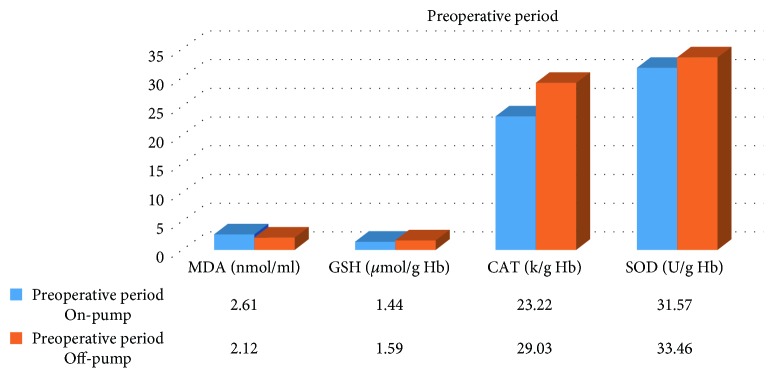
Statistical comparison of oxidative and antioxidant parameters in the on-pump and off-pump groups in the preoperative period.

**Figure 10 fig10:**
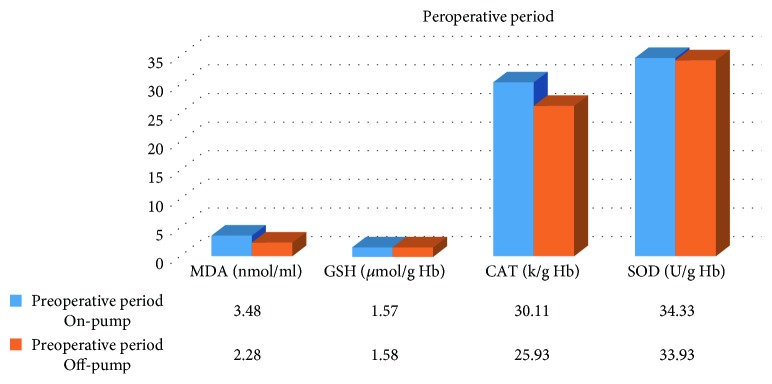
Statistical comparison of oxidative and antioxidant parameters in the on-pump and off-pump groups in the peroperative period.

**Figure 11:**
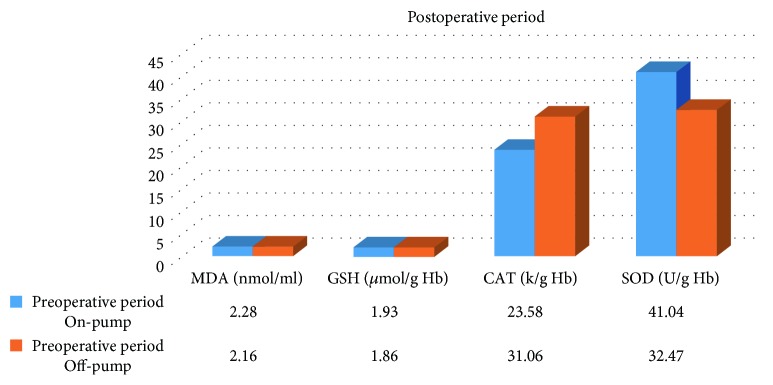
Statistical comparison of oxidative and antioxidant parameters in the on-pump and off-pump groups in the postoperative period.

A significant increase was determined in MDA level in the preoperative and peroperative periods in the on-pump group compared to the off-pump group, and no significant difference was observed in the postoperative period. No statistically significant difference was observed in GSH level and CAT activity in the preoperative, peroperative, and postoperative periods in the on-pump group compared to the off-pump group. While there was no significant difference in SOD enzyme activity in the preoperative and peroperative periods in the on-pump group compared to the off-pump group, a statistically significant increase was determined in SOD enzyme activity in the postoperative period in the on-pump group compared to the off-pump group (Table 3, Figures 9 [Fig fig10]–11).

No statistical significance was found in the biochemistry data of the on-pump and off-pump groups (Tables 4 and 5).

It is considered that oxidative stress is an important factor in the etiology and/or progression of many human diseases [22]. Several previous studies have shown the presence of oxidative stress in cardiopulmonary bypass and ischemia-reperfusion process [23, 24]. It is suggested that oxidative stress is responsible for the changes in myocardium during ischemia-reperfusion. Lipid peroxidation associated with the development of excessive reactive oxygen species in the cells leads to tissue damage as a result of protein denaturation, enzyme inactivation, and carbohydrate breakdown [25].

When the results of the present study were evaluated, it was found that in the preoperative period, MDA level was lower in the off-pump group than in the on-pump group.

The individuals in the off-pump group were the patients having relatively less incidence of coronary artery disease. While the mean number of artery grafts of the patients was 1.81, this number was 3.25 in the patients in the on-pump group and they were the patients having more common coronary artery disease. In the study of Cai et al., oxidative stress took a part in the pathogenesis of many cardiovascular diseases such as hypercholesterolemia, atherosclerosis, hypertension, diabetes, and cardiac failure [26]. Increased ROS production synthesized from dysfunctional endothelium and vascular smooth muscle cells affects a series of vascular structuring and causes vascular contraction by degrading the fluid dynamics and also atherosclerosis [27]. In the present study, it was also found that the MDA level was significantly lower in the preoperative period in the off-pump group with low mean graft number compared to the on-pump group. This result showed parallelism with these results.

In their study, Matata et al. [13] determined that lipid hydroperoxide level significantly increased between the 1st and 4th hours after the beginning of CABG. In the present study, a statistically significant increase was determined in MDA level in the peroperative period. This result was in parallel with these results. Also in the present study, the lack of a significant difference in the antioxidant parameters in the peroperative period compared to the preoperative period could not inhibit the increase in MDA. However, the fact that the increase in MDA level in the peroperative period was not observed in the postoperative period was thought to be associated with the increase in SOD activity in the postoperative period compared to the peroperative period and the significant increase in GSH level in the postoperative period compared to the preoperative period.

In the study of Gonenc et al., no significant difference was observed in the lipid peroxidation in the patients to whom OPCABG was applied [28]. In the present study, a statistically significant difference was not found in MDA and antioxidant parameters in the preoperative, peroperative, and postoperative periods. This result showed parallelism with the results of the study by Gonenc et al. [28]. This indicated that off-pump had no negative effect in formation of oxidative damage.

In their study, Gerritsen et al. determined that urinary lipid peroxidation level was lower in OPCABG compared to that in CABG [12]. Contrary to numerous studies [13], Gonenc et al. could not find a significant difference in the plasma lipid peroxide levels in their study when they compared OPCABG and CABG [28]. In the present study, a significant decrease was determined in MDA level in the preoperative and peroperative periods in the off-pump group compared to the on-pump group; the result was parallel with the results of the study by Gerritsen et al. [12]; however in parallel with the results of the study by Gonenc et al. [28], a significant difference was not found in MDA in the postoperative period between the off-pump and on-pump groups in the present study. These results showed that especially on-pump operation was effective in the formation of oxidative damage in the preoperative and peroperative periods.

It is known that the antioxidants in all the tissues act as a protector by creating an attack against free radicals. These antioxidants involve nonenzymatic antioxidants such as *α*-tocopherol and carotenoids that are responsible for extracellular defense and also antioxidant enzymes such as the SOD, GPx, and CAT that are responsible for intracellular defense. Myocardial antioxidants take a part in the inhibition of oxidative damage of lipid, DNA, protein, and carbohydrates [28]. Numerous studies have showed beneficial effects of antioxidants in order to protect the heart during ischemia-reperfusion damage [29]. In the present study, a significant increase was determined in SOD activity and GSH level in the on-pump group in the postoperative period. It was thought that these increases were effective in preventing the formation of lipid peroxidation.

Even if SOD activity significantly decreased in the postoperative period in the patients undergoing off-pump operation, the decrease only in SOD activity was not effective to trigger the increase in MDA level. Again in the present study, the absence of a significant difference in the antioxidant parameters in the preoperative and peroperative periods indicated that whether or not the pump was used had no significant effect on the activity of antioxidant parameters. The lack of a significant difference in antioxidant parameters in the preoperative and peroperative periods can be associated with the absence of an efficient defense mechanism upon the increase in MDA in the on-pump group.

Other studies revealed a significant decrease in total antioxidant capacity (TAC) level in the patients with CABG after the operation [30]. In another study, it was revealed that TAC level was lower in the patients with CABG compared to the patients with OPCABG [31]. The fact that the data of the present study did not show parallelism with the results of these studies was associated with the fact that in the present study, postoperative samples were taken approximately 24 hours later. Because in other studies specified, the decrease in TAC level was determined in the samples that were taken 72 hours after the operation.

Also, there was no statistical difference in demographic comparison. For this reason, we think that there is no effect of demographic findings on the presence of on-pump and off-pump oxidative damage that we aim to determine in our study.

## 4. Conclusions

The patients undergoing the on-pump operation were exposed to a more severe oxidative damage than the patients undergoing the off-pump operation, and we obtained the data indicating that on-pump operation is a method that triggers oxidative damage. However, it is known that on-pump surgical method is still a gold standard and we are of opinion that this method can be used when aorta cannulation and CPB method are contraindicated. Although it is specified that off-pump coronary artery surgery is disadvantageous than on-pump in terms of graft opening especially in long term, every patient should be assessed individually. It is thought that especially off-pump operation creates a disadvantage due to the formation of oxidative stress in early postoperative period in on-pump operations in which CPB is used, and in this case, off-pump operation may provide a serious benefit in elderly patient group in which coronary artery disease is less common. Also according to these data, we think that this oxidative damage can be suppressed by administering suitable antioxidant supplements in the preoperative and peroperative periods in patients undergoing on-pump operation. However, further clinical studies are required to investigate our opinion.

## Figures and Tables

**Table 1:** Statistical comparison of oxidative and antioxidant parameters in the on-pump group in the preoperative, peroperative, and postoperative periods.

Parameters	Preoperative period	Peroperative period	Postoperative period	*p*
MDA (nmol/ml)	2.61 ± 0.15^a^	3.48 ± 0.21^b^	2.28 ± 0.13^a^	*p* < 0.05
GSH (*μ*mol/g Hb)	1.44 ± 0.17^a^	1.57 ± 0.19^ab^	1.93 ± 0.20^b^	*p* < 0.05
CAT (k/g Hb)	23.22 ± 2.72^ab^	30.11 ± 2.90^a^	23.58 ± 3.38^b^	*p* < 0.05
SOD (U/g Hb)	31.57 ± 1.61^a^	34.33 ± 1.71^a^	41.04 ± 2.11^b^	*p* < 0.05

Letters a and b show statistically different groups.

**Table 2:** Statistical comparison of oxidative and antioxidant parameters in the off-pump group in the preoperative, peroperative, and postoperative periods.

Parameters	Preoperative period	Peroperative period	Postoperative period	*p*
MDA (nmol/ml)	2.12 ± 0.11	2.28 ± 0.18	2.16 ± 0.11	*p* > 0.05
GSH (*μ*mol/g Hb)	1.59 ± 0.21	1.58 ± 0.28	1.86 ± 0.30	*p* > 0.05
CAT (k/g Hb)	29.03 ± 3.69	25.93 ± 3.15	31.06 ± 3.18	*p* > 0.05
SOD (U/g Hb)	33.46 ± 1.87	33.93 ± 1.63	32.47 ± 1.41	*p* > 0.05

**Table 3:** Statistical comparison of oxidative and antioxidant parameters in the on-pump and off-pump groups in the preoperative, peroperative, and postoperative periods.

Parameters	Preoperative period	Peroperative period	Postoperative period
On/off-pump MDA (nmol/ml) *p*	*p* < 0.05	*p* < 0.05	*p* > 0.05
On/off-pump GSH (*μ*mol/g Hb) *p*	*p* > 0.05	*p* > 0.05	*p* > 0.05
On/off-pump CAT (k/g Hb) *p*	*p* > 0.05	*p* > 0.05	*p* > 0.05
On/off-pump SOD (U/g Hb) *p*	*p* > 0.05	*p* > 0.05	*p* < 0.05

**Table 4:** Demographic data of on-pump patients.

∑*n* 26	Groups	Urea	Creatinine	AST	ALT	CRP
Presence of Lima	Yes	33.90 ± 3.27	0.99 ± 0.10	26.45 ± 3.51	26.45 ± 4.34	7.78 ± 0.66
	No	40.26 ± 4.00	0.95 ± 0.50	20.80 ± 3.11	24.13 ± 3.77	7.31 ± 0.62
	*p*	*p* > 0.05	*p* > 0.05	*p* > 0.05	*p* > 0.05	*p* > 0.05

Presence of DM	Yes	42.66 ± 5.52	1.07 ± 0.09	22.00 ± 4.93	23.77 ± 4.96	7.57 ± 0.50
	No	34.88 ± 2.88	0.91 ± 0.06	23.82 ± 2.58	25.82 ± 3.48	7.47 ± 0.64
	*p*	*p* > 0.05	*p* > 0.05	*p* > 0.05	*p* > 0.05	*p* > 0.05

Presence of HT	Yes	38.26 ± 3.01	0.97 ± 0.58	23.30 ± 2.56	24.95 ± 2.91	7.27 ± 0.46
	No	32.33 ± 4.48	0.96 ± 0.31	22.33 ± 6.56	26.33 ± 11.46	9.32 ± 1.31
	*p*	*p* > 0.05	*p* > 0.05	*p* > 0.05	*p* > 0.05	*p* > 0.05

Smoking status	Smoking	35.45 ± 4.57	1.04 ± 0.62	23.54 ± 4.06	26.90 ± 5.25	7.52 ± 0.72
	Not smoking	39.13 ± 3.39	0.92 ± 0.76	22.93 ± 2.90	23.80 ± 3.08	7.50 ± 0.59
	*p*	*p* > 0.05	*p* > 0.05	*p* > 0.05	*p* > 0.05	*p* > 0.05

Presence of COPD	Yes	40.77 ± 6.81	1.06 ± 0.75	21.00 ± 4.93	25.22 ± 5.92	6.69 ± 0.77
	No	35.88 ± 2.20	0.92 ± 0.66	24.35 ± 2.55	25.05 ± 3.07	7.94 ± 0.54
	*p*	*p* > 0.05	*p* > 0.05	*p* > 0.05	*p* > 0.05	*p* > 0.05

**Table 5:** Demographic data of off-pump patients.

∑*n*	Groups	Urea	Creatinine	AST	ALT	CRP
26		(30.50 ± 2.59) (39.40 ± 2.31) *p*	(0.68 ± 0.23) (0.92 ± 0.21) *p*	(17.25 ± 2.01) (20.68 ± 3.59) *p*	(17.25 ± 1.25) (22.18 ± 2.59) *p*	(9.48 ± 2.56) (8.70 ± 1.67) *p*
Presence of DM	Yes	0.125	0.213	0.189	0.224	0.682
	No	*p* > 0.05	*p* > 0.05	*p* > 0.05	*p* > 0.05	*p* > 0.05

Presence of HT	Yes	0.568	0.492	0.087	0.489	0.321
	No	*p* > 0.05	*p* > 0.05	*p* > 0.05	*p* > 0.05	*p* > 0.05

Smoking status	Smoking	0.705	0.867	0092	0.152	0.457
	Not smoking	*p* > 0.05	*p* > 0.05	*p* > 0.05	*p* > 0.05	*p* > 0.05

Presence of COPD	Yes	0.055	0.138	0.271	0.656	0.816
	No	*p* > 0.05	*p* > 0.05	*p* > 0.05	*p* > 0.05	*p* > 0.05
